# Protein sociology of ProA, Mip and other secreted virulence factors at the *Legionella pneumophila* surface

**DOI:** 10.3389/fcimb.2023.1140688

**Published:** 2023-03-02

**Authors:** Lina Scheithauer, Mustafa Safa Karagöz, Benjamin E. Mayer, Michael Steinert

**Affiliations:** ^1^ Institut für Mikrobiologie, Technische Universität Braunschweig, Braunschweig, Germany; ^2^ Computational Biology & Simulation, Technische Universität Darmstadt, Darmstadt, Germany

**Keywords:** Legionella pneumophila, surface-associated proteins, secreted effectors, zinc metalloprotease ProA, macrophage infectivity potentiator, interactomics, computational biology

## Abstract

The pathogenicity of *L. pneumophila*, the causative agent of Legionnaires’ disease, depends on an arsenal of interacting proteins. Here we describe how surface-associated and secreted virulence factors of this pathogen interact with each other or target extra- and intracellular host proteins resulting in host cell manipulation and tissue colonization. Since progress of computational methods like AlphaFold, molecular dynamics simulation, and docking allows to predict, analyze and evaluate experimental proteomic and interactomic data, we describe how the combination of these approaches generated new insights into the multifaceted “protein sociology” of the zinc metalloprotease ProA and the peptidyl-prolyl *cis/trans* isomerase Mip (macrophage infectivity potentiator). Both virulence factors of *L. pneumophila* interact with numerous proteins including bacterial flagellin (FlaA) and host collagen, and play important roles in virulence regulation, host tissue degradation and immune evasion. The recent progress in protein-ligand analyses of virulence factors suggests that machine learning will also have a beneficial impact in early stages of drug discovery.

## 1 Introduction

Legionellosis emerged in the second half of the 20th century as a consequence of engineered warm-water habitats, which enable increased reproduction of *Legionella pneumophila* and efficient transmission of pathogen-containing aerosols to humans (McDade et al., 1979; Breiman, 1996; Albert-Weissenberger et al., 2007; Campbell and Cianciotto, 2022). Hot water facilities such as cooling towers, whirlpools, hot tubs, or showers are sources and technical vectors of infection, since natural freshwater reservoirs usually contain lower bacterial densities (Jernigan et al., 1996; Keller et al., 1996; Atlas, 1999; Fields et al., 2002; Inoue et al., 2015; Molina et al., 2022).

*L. pneumophila* is a Gram-negative, obligate aerobic and rod-shaped bacterium from the ɣ-Proteobacteria class. In its natural habitat, replication occurs intracellularly within protozoa, especially amoebae of the genus *Acanthamoeba, Naegleria* and *Vermamoeba* (Rowbotham, 1980; Watanabe et al., 2016; Nisar et al., 2020; Price and Abu Kwaik, 2021). Due to improved diagnostics, as well as demographic and climatic changes, the number of cases of legionellosis continues to rise (Fischer et al., 2020; de Jong and Hallström, 2021; Portal et al., 2021; Barskey et al., 2022). Thus, *Legionella* became the most significant waterborne pathogen in terms of its spread and the severity of infection (Neil and Berkelman, 2008; Walker, 2018). Evoked respiratory diseases are differentiated between the self-limiting Pontiac fever with flu-like symptoms and the life-threatening Legionnaires’ disease (Fraser et al., 1977; Glick et al., 1978).

Legionnaires’ disease is a multisystemic form of legionellosis associated with atypical pneumonia. It is caused by bacterial proliferation predominantly in alveolar macrophages but also in lung epithelial cells, which results in necrotic damage to the pulmonary tissue (Horwitz and Silverstein, 1980; Blackmon et al., 1981; Mody et al., 1993; Cianciotto et al., 1995). Risk groups of Legionnaires’ disease especially include elderly, males, smokers and patients with immunosuppressive diseases or treatments (Hoge and Breiman, 1991; Marston, 1994; Neil and Berkelman, 2008). The mortality rate varies considerably depending on risk factors, the source of infection and antibiotic therapy. About one out of ten patients dies from respiratory failure (Buchholz et al., 2010).

The pathogenicity of *L. pneumophila* for humans is considered to be a result of its co-evolution with protozoa pre-adapting the bacterium to phagocytes and likewise enabling its replication within human alveolar macrophages. The infection of these evolutionarily distant hosts shows strong similarities and is based on the pathogen’s ability to inhibit phagolysosomal degradation (Horwitz, 1983; Bozue and Johnson, 1996; Gao et al., 1997; Segal and Shuman, 1999; Albert-Weissenberger et al., 2007). While other bacterial species undergo enzymatic digestion after acidification, the *Legionella*-containing vacuole (LCV) matures into an ER-like (endoplasmic reticulum) compartment with rather neutral pH values (Swanson and Isberg, 1995; Wieland et al., 2004). Although conservation of intracellular, eukaryotic signaling pathways enables the bacterium to multiply in our lungs, it is not adapted to humans as hosts, since further transmission from person to person does not take place (Abu Kwaik, 1996; Molofsky and Swanson, 2004).

*L. pneumophila* shows a biphasic life cycle switching between a replicative, intracellular and a transmissive, infectious state. In this context, expression of important virulence traits and flagellation are closely linked to the transmissive, stationary growth phase, when *L. pneumophila* requires access to new host cells (Swanson and Hammer, 2000; Molofsky and Swanson, 2004; Chauhan and Shames, 2021). For the infection of many different host species, *L. pneumophila* possesses an exceptionally versatile spectrum of virulence factors. Remarkably, the bacterium acquired numerous eukaryotic-like genes or domains *via* horizontal gene transfer from its protozoan hosts (Brenner et al., 1979; Cazalet et al., 2004; Mondino et al., 2020a; Hilbi and Buchrieser, 2022). Specific interactions in mammals, such as the manipulation of the NF-κB signaling pathway, further suggest that simple environmental metazoans also played a role in the evolution of *L. pneumophila*. These virulence factors are key elements for influencing host cell trafficking and immune evasion, and thus pre-adapt and enable the pathogen to evoke severe pneumonia in humans (Cazalet et al., 2004; Steinert et al., 2007; Lurie-Weinberger et al., 2010; Best and Abu Kwaik, 2018). In contrast to amoebal infections, the life cycle in higher organisms additionally requires extracellular pathogenicity for tissue dissemination and protection from the host response. This is primarily mediated by surface proteins and secreted effectors, which are of high importance during Legionnaires’ disease.

In this review, we focus on secretory and surface-associated virulence proteins of *L. pneumophila* and their respective interactions with each other as well as with host structures. We describe how bacterial effector secretions reprogram host cells and determine the pathogenicity of Legionnaires’ disease. The major secreted protease ProA and the membrane-associated peptidyl-prolyl *cis/trans* isomerase (PPIase) Mip of *L. pneumophila* will be especially highlighted, since they exhibit a multifaceted “protein sociology” with bacterial and host interaction partners. We discuss overlapping target structures of ProA and Mip like bacterial flagellin and the host ECM protein collagen and describe how the combination of X-ray structures, interactomic approaches and modern computational biology methods were used for simulation and exploration of biochemical processes at the pathogen-host interface. Moreover, we will briefly outline potentials related to the use of computational biology in drug discovery.

## 2 Surface-associated bacterial proteins target extra- and intracellular host proteins and contribute to different phases of infection

Proteins on the cell surface of *L. pneumophila* play a central role for pathogen-host interactions (**Table 1**). In the case of macrophages, recognition, adherence and complement-mediated phagocytosis of *L. pneumophila* takes place *via* the host cell receptors CR1 and CR3. The major outer membrane protein MOMP serves as the interacting acceptor molecule on the bacterial site (Horwitz and Silverstein, 1981; Payne and Horwitz, 1987; Bellinger-Kawahara and Horwitz, 1990). However, complement-independent attachment is likewise possible *via* type IV pili. Those are expressed by the pathogen in different lengths from 0.1 to 1.5 µm. Adherence to different host cells is only mediated by longer forms with at least 0.8 µm, for which a functional *L. pneumophila* pilin pilE_L_ is necessary. They are also involved in the colonization of biofilms or human lung tissue, as well as the invasion of free-living amoebae (Stone and Abu Kwaik, 1998; Lucas et al., 2006). Gene expression and pili biogenesis are increased at 30°C compared to 37°C and correlate with the natural competence of *L. pneumophila*, hence promoting genomic recombination with transformed DNA. Therefore, these pili are referred to as CAP for competence and adherence associated pili (Liles et al., 1998; Stone and Kwaik, 1999). Studies have shown that the membrane-localized protein PilY1, which is involved in type IV pili biogenesis, accordingly represents a virulence factor affecting twitching motility, adherence and invasion but also replication of the pathogen (Hoppe et al., 2017).

**Table 1:** Surface-associated and secreted proteins of *L. pneumophila* and their extra- and intracellular host targets addressed in this review.

Factor	Protein type	Host target	Activity and function	Reference
Surface proteins				
FlaA	Flagellin	Targeted by TLR5, Naip5/Nlrc4	Immunogenic properties, bacterial motility and invasion	Dietrich et al., 2001
MOMP	Major outer membrane protein	CR1 and CR3	Cell entry, acceptor molecule for complement-mediated phagocytosis	Bellinger-Kawahara and Horwitz, 1990
Mip	Peptidyl-prolyl *cis/trans* isomerase	Collagen I-VI	Moonlighting activities, contributes to infection, transmigration, and colonization of nematodes	Karagöz et al., 2022
PilD	prepilin peptidase		Promotes pilus biogenesis, protein secretion and intracellular growth	Rossier and Cianciotto, 2001
pilE_L_	Pilin		Associated with bacterial adherence, colonization, invasion	Stone and Abu Kwaik, 1998
PilY1	Type IV pili biogenesis protein		Promotes twitching motility, adherence, invasion and replication	Hoppe et al., 2017
TolC	Efflux pump		Stress resistance, important during early phase of host cell infection	Ferhat et al., 2009
Secreted proteins				
AnkB	F-box protein	E3 ubiquitin ligase, Parvin B	Promotes proteasomal degradation, essential for intracellular life cycle	Richards et al., 2013
ChiA	Endochitinase	MUC5AC, C1-INH	Mucinase activity, survival and transmigration in human lungs	Rehman et al., 2020
DotA	T4SS component		Effector secretion, promotes intracellular replication	Nagai and Kubori, 2011
LapA	M28 peptidase	Aminopeptides	Nutrient acquisition, promotes infection of *A. castellanii*	White et al., 2018
LipA	Lipase	Mono- and triacylglycerols	Lipolytic activity	Aragon et al., 2002
LpnE	SLR protein	OBSL1	Cell entry, trafficking of the LCV	Newton et al., 2007
NttA	T2SS substrate	Phosphoinositides like PtdIns(3,5)P_2_	Association with the LCV, replication in *A. castellanii*	Portlock et al., 2020
PlaA	GDSL lipase/acyltransferase	Cholesterol, ergosterol, lysophospholipids	Major LPLA activity, transfers propionic acid, promotes LCV destabilization for bacterial exit	Lang et al., 2017
PlaC	GDSL lipase/acyltransferase	Cholesterol, ergosterol, diacylphospholipids	Major PLA and GCAT activity, transfers palmitic acid, promotes growth within amoebae	Lang et al., 2012
PlcA	Phospholipase C		Catalyses pNPPC hydrolysis	Aragon et al., 2002
ProA	M4 zinc metalloprotease	Various, e.g., TNF-α, collagen IV, IL-2, IL-6, CD4	Hemolytic and cytotoxic, tissue degradation, intracellular growth, transmigration and immune evasion	Scheithauer et al., 2021
SdhA	T4SS substrate	OCRL protein	Promotes LCV integrity, evasion of death pathways and intracellular growth in macrophages	Choi et al., 2021
SrnA	RNase		Replication in *V. vermiformis* and *N. lovaniensis*	Tyson et al., 2013

Even though alveolar macrophages feature the main reservoir of *L. pneumophila* in human lungs, the pathogen is also able to infect pulmonary epithelial cells, which makes invasion strategies more important. The surface protein and chaperone Hsp60 of the GroEL family is able to promote the entry into those non-phagocytic host cells and, moreover, induces the establishment of the LCV. It is especially synthesized in the early phase of infection and secreted into the phagosome, leading to the recruitment of mitochondria and increased cytokine expression in macrophages (Fernandez et al., 1996; Garduño et al., 1998; Chong et al., 2009; Zhan et al., 2015). Factors which play a role in cell entry and manipulation of the intracellular transport often contain the predominantly eukaryotic SLR motif (Sel1 repeats) for protein-protein interactions (Newton et al., 2007). Examples are LpnE and the periplasmic EnhC, which additionally influences survival under H_2_O_2_ stress, cell integrity and immune evasion (Liu et al., 2008; Liu et al., 2012).

As a Gram-negative bacterium, *L. pneumophila* has the ability to release effectors in so-called extracellular vesicles (EVs), which contain a variety of immunomodulatory proteins and display surface-associated proteins and lipopolysaccharides (LPS) on the outside. They are used for communication between bacteria, but can also fuse with host cells and transmit virulence factors including RNA (Sahr et al., 2022). In this way, EVs cause lung tissue damage and provide replication advantages for the pathogen (Jäger et al., 2015). Various effectors such as the chitinase ChiA, the macrophage infectivity potentiator Mip, the zinc metalloprotease ProA and LPS can be components of EVs (Galka et al., 2008). The LPS of *L. pneumophila* differs significantly from that of other bacteria due to its composition and low endotoxic potential. The membrane-bound lipid A exhibits particularly long-chain and branched fatty acids. Therefore, LPS can act as an adhesion factor that anchors the bacterium in the host cell membrane (Zähringer et al., 1995; Neumeister et al., 1998). In addition, it allows evasion of the pathogen from lysosomal digestion (Seeger et al., 2010). Depending on the phase of infection, *L. pneumophila* is able to express two different forms of LPS. A more hydrophilic variant facilitates the close spatial proximity of *Legionella* in the phagosome, while a hydrophobic form facilitates adhesion to host cell membranes, as well as survival and transmission of the bacterium in aerosols (Reichardt et al., 2010; Palusinska-Szysz et al., 2019). Although Mip is also an EV-associated surface protein, it does not play a role in bacterial adherence to host cells, but rather confers resistance of the pathogen to intracellular digestion by the host (Cianciotto et al., 1990; Cianciotto and Fields, 1992). The FK506-binding protein is of decisive importance for the proliferation rate of *L. pneumophila* in protozoa and macrophages. Its particular role for Legionnaires’ disease will be discussed in a later chapter of this review.

For motility in the transmissive phase, *L. pneumophila* possesses a single monopolar flagellum on its cell surface, which also plays a crucial role in the establishment of infection. Over 50 different genes are involved in its functional expression assembling a membrane-spanning basal body, a hook and an extracellular filament (Chilcott and Hughes, 2000). On the cytoplasmic side, the motor complex MotAB generates the required rotational energy *via* proton motive force. Extracellular components are secreted by a type III-like export apparatus. The filament itself is formed by polymerization of the 48 kDa subunit FlaA, which is, assisted by FliD, gradually incorporated at the tip (Altegoer et al., 2014; Appelt and Heuner, 2017).

Transcription of the flagellin gene *flaA* is temperature-dependently regulated by the alternative sigma factor σ^28^ FliA (Heuner et al., 1995). Thus, FlaA expression and motility are primarily determined by the growth phase, but also by environmental factors such as the availability of amino acids and the viscosity or osmolarity of the medium (Heuner et al., 1999). FliA itself is part of the post-exponential regulatory cascade triggered by the alarmon ppGpp in response to nutrient limitation. At this point of the *L. pneumophila* life cycle, the flagellum facilitates access to new host cells and increases the rate of invasion. Therefore, proliferation of a *flaA* deletion mutant is attenuated in amoebae and human cell lines (Dietrich et al., 2001; Teruya et al., 2007). However, free, unassembled flagellin induces the innate immunity of alveolar macrophages as it is recognized by various host receptors such as Toll-like receptor 5 (TLR5) on the cell surface (Palusinska-Szysz and Janczarek, 2010). TLR5 is of great importance regarding the bacterial clearance or susceptibility towards Legionnaires’ disease and regulates, for example, the recruitment of polymorphonuclear neutrophils (PMNs) into the alveolar space (Hawn et al., 2007). After stimulation of the receptor TLR5, the adapter molecule Myd88 (myeloid differentiation primary response 88) is induced first. The signal is further transmitted *via* the IκB kinase IKK and the mitogen-activated protein kinase p38 MAPK (Yu et al., 2003). As a result of phosphorylation, the inhibitor IκB is degraded and its target NF-κB is released. The activation of the transcription factor NF-κB finally leads to the expression or secretion of immunoregulatory factors such as tumor necrosis factor-α (TNF-α), interleukin-8 (IL-8) and IL-6, as well as other chemokines. A nonsense mutation in the TLR5 gene, therefore, significantly increases susceptibility to *L. pneumophila* infections (Hawn et al., 2003; Hawn et al., 2007). Furthermore, it was shown that flagellin monomers are occasionally translocated into the host cell *via* the bacterial Dot/Icm type IV secretion system (T4SS) (Fontana and Vance, 2011). On the cytosol side, nucleotide-binding oligomerization domain (NOD)-like receptors (NLRs) such as neuronal apoptosis inhibitory protein 5 (Naip5) and the NLR family CARD domain-containing protein 4 (Nlrc4, formerly: Ipaf) are stimulated in a flagellin-dependent manner. These in turn can prevent the proliferation of *Legionella* in murine macrophages by activating caspase-1 and, moreover, contribute to the maturation of inflammatory cytokines such as IL-1β and IL-18. Naip5 induces early apoptosis, whereas binding of Nlrc4 leads to lysosomal digestion of the bacteria (Amer et al., 2006; Ren et al., 2006; Lamkanfi et al., 2007). Akhter et al. showed that the activation of caspase-7 plays a crucial role in these processes as well (Akhter et al., 2009). Both NLRs together form the Naip5/Nlrc4 inflammasome, which ultimately leads to pyroptosis, a rare form of cell death associated with the initiation of a severe inflammatory response (Kay et al., 2020; Chauhan and Shames, 2021). The proliferation of *L. pneumophila* is effectively limited by intact NLRs in mice making them resistant against Legionnaires’ disease. The human orthologues also mediate immune defense against flagellin-expressing bacteria, however, the host cells can still be infected and severe courses of the disease occur (Vinzing et al., 2008).

## 3 Type I, II and IV effector secretion orchestrates host cell manipulation and increases the outreach of protein interactions in tissue

Selection for survival in the environment and intracellular replication in diverse protists has resulted in the accumulation of a broad arsenal of effectors by *Legionella* (**Table 1**). Thus, besides an interaction of the pathogen with host cells *via* membrane-bound surface proteins, *L. pneumophila* is most notably able to secrete a versatile repertoire of virulence factors to the extracellular space or directly into the host cell cytosol (White et al., 2019; Costa et al., 2021). Accordingly, the bacterium features different secretion systems that are fundamental to cellular and tissue pathogenesis.

The type I secretion system (T1SS), encoded by the lssXYZABD locus, plays also an important role regarding the host cell entry and is involved in sliding motility of the pathogen (Jacobi and Heuner, 2003; Fuche et al., 2015). One of its central components represents the membrane protein TolC, which acts as an efflux pump contributing to the early phase of invasion and intracellular replication (Ferhat et al., 2009). Even though it is not able to directly inject effectors into the host cell cytoplasm, the T1SS plays a major role for the intracellular life cycle in various amoeba species, alveolar macrophages as well as lung epithelial cells. It contributes to the persistence of the pathogen in lung tissue and attenuates the host’s chemokine and cytokine response (McCoy-Simandle et al., 2011; Cianciotto et al., 2013; Mallama et al., 2017). Additionally, it regulates growth of *L. pneumophila* at low temperatures as well as biofilm formation (Söderberg et al., 2004; White and Cianciotto, 2019).

Like the T1SS, the type II secretion system (T2SS) lsp (*Legionella* secretion pathway) is involved in the secretion of surfactants for sliding motility. These surfactants additionally exhibit antibacterial properties and create a selective advantage for *L. pneumophila* over other *Legionella* species (Stewart et al., 2011). The T2SS is built up from the membrane proteins LspD in the inner membrane, LspF in the outer membrane, and the ATPase LspE. It is also dependent on the protein PilD, which is essential not only for the biogenesis of type IV pili but for the processing of the pseudopilins LspG-K (Liles et al., 1998; Rossier and Cianciotto, 2001). Studies have shown that some T2SS substrates first utilize the twin-arginine translocation (Tat) system of the inner membrane for export to the periplasm and additionally require the lsp system for final secretion (Rossier and Cianciotto, 2005). The T2SS translocates over 25 effector proteins such as the M4 zinc metalloprotease ProA, phospholipases A and C, RNAses, and NttA-E, which are essential for optimal infection of different amoeba species (Rossier et al., 2009; Tyson et al., 2013; Portlock et al., 2020). In this context, the protease ProA was the first discovered substrate of a T2SS which is involved in the intracellular infection of a bacterial pathogen (Hales and Shuman, 1999; Rossier et al., 2004). The tissue destructive protease not only promotes replication in several amoebal hosts but also determines extracellular pathogenicity during Legionnaires’ disease (Moffat et al., 1994a; Edelstein et al., 1999; Tyson et al., 2013).

The most important secretion system, which is essential for the whole life cycle of *L. pneumophila*, is the Dot/Icm type IVB secretion system (T4SS) (Lockwood et al., 2022). Translocated effector proteins control the bacterial uptake and egress, disrupt the phagolysosome fusion, and manipulate many critical processes in the eukaryotic cell, such as vesicle transport, signal transduction, gene expression and protein translation, ubiquitination, cytoskeleton dynamics, autophagy, apoptosis pathways, and host defense (Berger and Isberg, 1993; Segal et al., 2005; Michard and Doublet, 2015; Hilbi et al., 2017; Allgood and Neunuebel, 2018; Schuhmacher et al., 2018; Yu et al., 2018; Gomez-Valero et al., 2019; Kitao et al., 2020). Interestingly, T4SS effectors have recently been shown to reverse the activity of mitochondrial FoF_1_-ATPase to ATP-hydrolysis, which even enables the pathogen to precisely time host cell death (Escoll et al., 2021; Kubori et al., 2022). Moreover, bacterial effectors not only mediate post-translational modifications of eukaryotic proteins but also are able to enter the host cell nucleus for a direct upregulation of transcription (Schuelein et al., 2018).

With more than 300 substrates the Dot/Icm type IVB secretion system secretes the largest repertoire of virulence-associated effectors of any pathogen species known to date (Al-Quadan et al., 2012; Lockwood et al., 2022). They enable the bacterium to infect many hosts from different phyla and with evolutionary distance (Boamah et al., 2017; Graells et al., 2018; Moreno et al., 2019). In fact, over 18 000 putative Dot/Icm substrates have been identified across the genus *Legionella*, which is known for the additional expression of so-called metaeffectors. This type of effectors represents a rather unique level of regulation since it is able to target other bacterial effectors and control their activity *via* different modes of action such as degradation or activation (Kubori et al., 2010; Gomez-Valero et al., 2019; Mondino et al., 2020b; Joseph and Shames, 2021). A large number of T4SS substrates also exhibits functional redundancy, which effectively protects the pathogen from a defect in intracellular replication (Ninio and Roy, 2007; Isberg et al., 2009). Substrate recognition and secretion occurs *via* the membrane-bound T4 coupling complex (T4CC). This is assembled from a DotLMNYZ core with DotL as the ATPase and the chaperones IcmSW (Meir et al., 2020). DotA of the inner membrane is essential for effector secretion and in turn for the intracellular proliferation of *L. pneumophila*. Its own secretion *via* the T4SS and the high recombination rate suggest direct interaction with the host. However, the exact function of DotA has not yet been clarified (Nagai and Kubori, 2011; Gomez-Valero and Buchrieser, 2019).

## 4 Major secretory protein ProA

### 4.1 ProA promotes infection of certain cell types and contributes to lung tissue destruction

ProA was first described in 1981 by Thompson et al. analyzing the *L. pneumophila* serogroups 1-6 (Thompson et al., 1981). Continuative studies regarding the distribution of different T2SS effectors in the genus *Legionella* revealed that ProA occurs as one of only two core substrates in all 57 species tested (White and Cianciotto, 2019). On the whole, *L. pneumophila* codes for various proteases, some of them annotated as homologues of other pathogens suggesting important virulence-associated functions. According to the MEROPS peptidase database, a total of 188 confirmed or putative proteases are listed for the bacterium, 57 of them are classified as zinc metalloproteases (www.ebi.ac.uk/merops/). In this context, ProA is the most important and best-characterized representative in *L. pneumophila*. The M4 enzyme was originally referred to as the major secretory protein (Msp), since it is the most abundant protein in the supernatant of *Legionella* cultures (Blander et al., 1990).

ProA does not appear to hold a universal function during intracellular proliferation of *L. pneumophila*, comparing different environmental hosts and infected cell types. Infection studies with amoeba strains of natural aquatic habitats revealed importance of ProA for optimal proliferation in *Vermamoeba vermiformis* and *Naegleria lovaniensis*, with a variety of tasks in *N. lovaniensis* and activation of bacterial PlaC as the key feature in *V. vermiformis*. In terms of *Acanthamoeba castellanii*, experiments from 2013 did not reveal any biological relevance for infection. Regarding the initiation phase, this was also attested in recent experiments from 2019 (Rossier et al., 2008; Tyson et al., 2013; White et al., 2019). Nevertheless, as replication progresses, a *proA*-deficient mutant showed reduced growth rates with lower cell counts compared to the *Legionella* wild type. Due to its extracellular enzymatic activity, ProA might promote, together with other T2SS effectors, the accessibility of amino acids, especially important in later stages of infection (Rossier et al., 2008; White et al., 2019).

**Figure 1:**
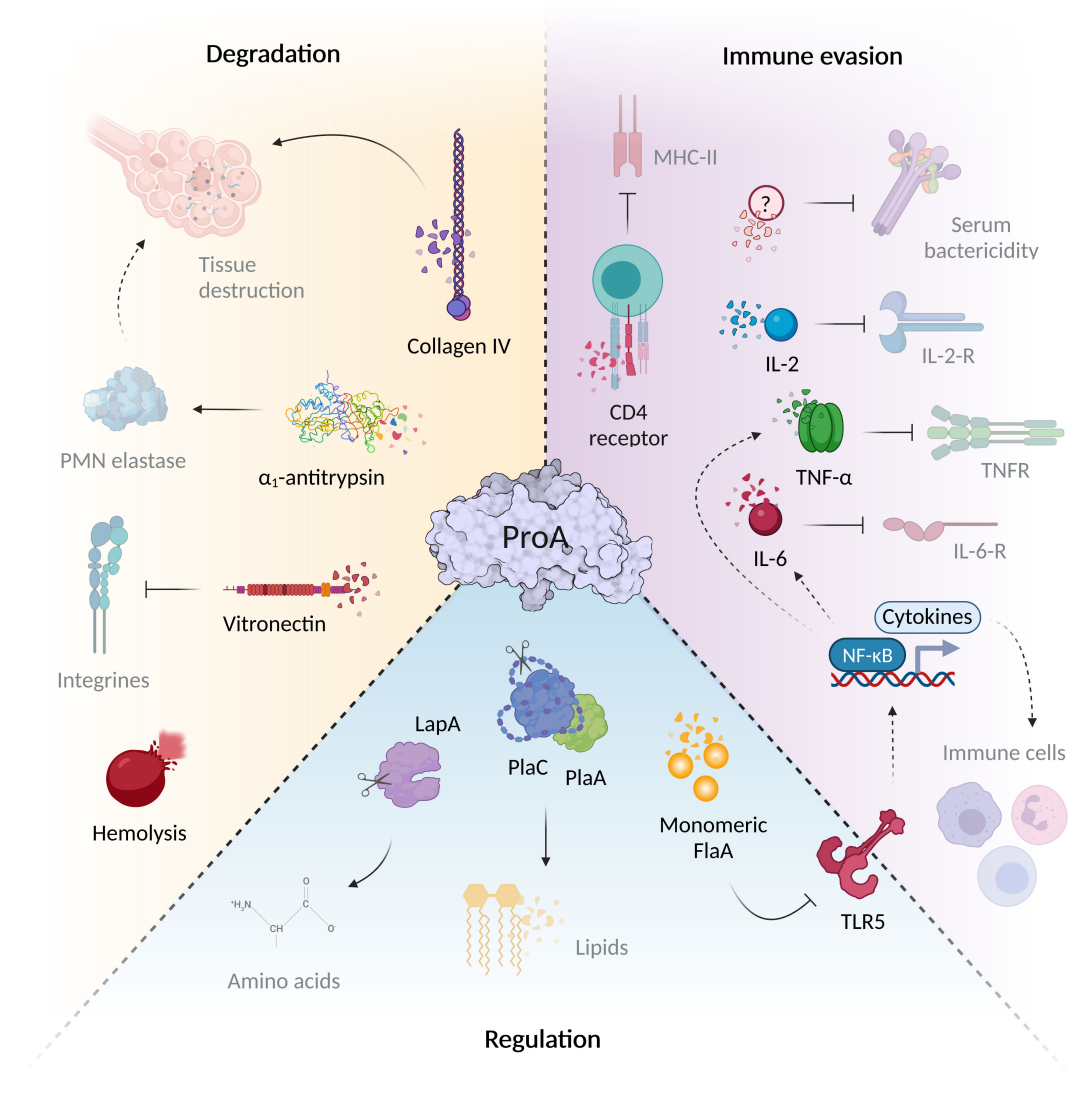
ProA mediates virulence regulation, host tissue degradation and immune evasion. The virulence factor ProA can interfere with a wide variety of infection-associated processes *via* the diverse range of substrates. During Legionnaires’ disease, it significantly mediates tissue degradation due to structural host targets such as collagen IV of the basal lamina and the cell adhesion protein vitronectin. ProA also cleaves α_1_-antitrypsin, important for controlling of host proteases. In addition, ProA exhibits hemolytic properties and contributes significantly to immune evasion. The zinc metalloprotease not only cleaves humoral serum components and chemokines (IL-2, IL-6, TNF-α), but also influences the recruitment and proliferation of various immune cells *via* these signaling molecules of specific surface receptors. However, ProA also attacks cellular structures directly, for example, by degrading the CD4 receptor. Aside from host targets, the protease also affects other bacterial proteins that play an important role during the intracellular life cycle of *L. pneumophila*. *Via* cleavage of exogenous flagellin, the TLR5-mediated immune response and thus the cytokine expression are reduced. Through direct processing, ProA can additionally activate virulence factors such as PlaA/PlaC and LapA, which contribute to lipid degradation at the LCV and amino acid synthesis (created with BioRender.com).

In addition to the life cycle in natural environments, early experiments in guinea pigs also pointed towards importance of ProA as a virulence factor for Legionnaires’ disease. Thus, intranasal inoculation with purified protease caused inflammation and lung lesions with alveolar hemorrhage, edema, tissue necrosis, and infiltration of PMNs and macrophages. These histomorphological changes closely resembled findings of a real *L. pneumophila* infection and were associated with the localization of ProA (Baskerville et al., 1986; Conlan et al., 1986; Williams et al., 1987). Moreover, increased humoral and cellular immunity of guinea pigs could be demonstrated after subcutaneous administration of the protease (Blander and Horwitz, 1989; Blander et al., 1990). Infections with a *proA*-negative mutant, however, led to contradictory results in animal models. Early studies in this context have not been able to display virulence-associated functions of the protease (Blander et al., 1990). Later, influence of ProA on bacterial replication, the course of disease, and necrosis of pulmonary tissue was described in guinea pigs (Moffat et al., 1994a; Edelstein et al., 1999). Forty years after discovery, the role of ProA was specified recently in human lung tissue explants (HLTEs) using purified protease as well as *proA* mutant strains. This detailed elucidation evidenced protease-dependent tissue destruction with swelling and disintegration of alveolar septa as well as increased bacterial proliferation, transmigration, and immune evasion of ProA-expressing *Legionella* strains in the physiological human background (**Figure 1**) (Scheithauer et al., 2021; Scheithauer et al., 2022).

Regarding monocellular systems, ProA could be detected intracellularly in amoebae and alveolar macrophages of guinea pigs or humans 24 h post infection (Rechnitzer and Kharazmi, 1992; Moffat et al., 1994a). Interestingly, the zinc metalloprotease was shown to be translocated into the host cell cytoplasm and, after 6 h, accumulates on the surface of the LCV. In this context, the first evidence for a T4SS-independent cytosol transport was found indicating semi-permeability of the LCV membrane (Truchan et al., 2017). Although ProA is produced during intracellular replication in multicellular hosts, no relevance to bacterial growth or host cell death could initially be demonstrated in macrophages (Szeto and Shuman, 1990; Moffat et al., 1994a; Rossier et al., 2008). However, subsequent analyzes by Edelstein et al. indicated diminished proliferation of the *proA*-negative *L. pneumophila* mutant in macrophages as well as lungs of guinea pigs (Edelstein et al., 1999). Analogously, slightly reduced bacterial replication was revealed in human cell lines, especially in A549 lung epithelial cells (Scheithauer et al., 2021). Attenuation after *proA* deletion can therefore be partly attributed to a restricted intracellular proliferation. However, epithelial cells, though susceptible, do not represent the preferred reservoir of *Legionella* in human lungs (Jäger et al., 2014). Phenotypes and histological observations regarding to ProA are therefore assumed to mainly result from extracellular activity of the secreted protease, for example, against structural host tissue factors.

The zinc metalloprotease ProA possesses an extraordinarily broad and diverse spectrum of substrates, and displays hemolytic as well as cytotoxic activity (**Figure 1**) (Conlan et al., 1986; Keen and Hoffman, 1989; Quinn and Tompkins, 1989). Despite highest sequence homology to the elastase pseudolysin from *Pseudomonas aeruginosa* ProA is not able to cleave human elastin fibers. Nevertheless, structural proteins such as casein, gelatin and collagen are among the known substrates (Thompson et al., 1981). Many M4 proteases function as key factors for pathogenesis, since they degrade targets with important roles during infection. The versatile spectrum of ProA substrates comprises human host proteins as well as bacterial effectors (**Figure 1**). By proteolysis of structural and immunomodulatory compounds, ProA provokes tissue degradation and bacterial evasion, especially important in infection of higher organisms. Furthermore, it can influence various processes of the cellular life cycle by regulating other virulence factors of *L. pneumophila*. Truchan et al. revealed that ProA associates in a ring-like structure along the outer leaflet of the LCV membrane enabling direct interaction with proteins of the host cell cytoplasm (Truchan et al., 2017). Presumably, ProA anchors itself *via* a putative farnesylation domain at the C-terminus and cleaves host factors to influence downstream signaling pathways. It is also conceivable that ProA contributes to the acquisition of nutrients for the proliferation of *L. pneumophila*, thus, supporting known mechanisms of the virulence factor AnkB, which exploits the ubiquitination machinery of the host cell (Richards et al., 2013; Truchan et al., 2017; White et al., 2018).

### 4.2 ProA targets host immunity and degrades human collagen for tissue transmigration

Substrates of the zinc metalloprotease ProA comprise various host proteins, which enable the enzyme to intervene in different areas of pathogenesis (**Figure 1**). Outside its protective intracellular replication niche, *L. pneumophila* needs to fight the host immune system. Accordingly, extracellular proteases like ProA can act as a first line of defense against humoral or cellular immune factors to inhibit both innate and acquired defense mechanisms. Early studies in 1980 demonstrated a direct cleavage of serum proteins by the bacterial metalloprotease (Müller, 1980). ProA might represent, together with LPS and ChiA, one of the pivotal factors to promote serum resistance of the pathogen and its protection from complement-mediated lysis (Khan et al., 2013; Rehman et al., 2020; Scheithauer et al., 2021). With regard to the bactericidal effect, factors of the complement system function as important serum components. They not only mediate direct lysis, but also contribute to the removal of the pathogen by phagocytic immune cells *via* opsonization. Among different *L. pneumophila* strains, high resistance to complement factors is a characteristic trait of virulent in contrast to non-virulent strains (Plouffe et al., 1985; Caparon and Johnson, 1988). Purified ProA is able to degrade, for example, the human serum protein and important inhibitor α_1_-antitrypsin, which controls activity of host proteases in pulmonary tissue (Conlan et al., 1988). It is therefore conceivable that *L. pneumophila* pursues a strategy similar to *P. aeruginosa* causing substantial destruction of lung tissue by endogenous serine proteases *via* M4 enzyme activity (Morihara et al., 1979). Another humoral *in vitro* substrate of ProA and well-known pro-inflammatory cytokine is TNF-α, which limits intrapulmonary replication of the pathogen in the early phase of infection (Hell et al., 1993; McCoy-Simandle et al., 2011). It is mainly released by macrophages and important for the PMN-mediated controlling of legionellosis (Blanchard et al., 1988). *L. pneumophila* mutant strains lacking the ProA-secreting T2SS showed significantly enhanced levels of TNF-α in infected U937 cells or mice lungs (McCoy-Simandle et al., 2011). Regarding the cellular immune response, ProA was shown to be able to impair the chemotaxis of PMNs and monocytes in a concentration-dependent manner, to impede the production of reactive oxygen species, and thus bacterial elimination *via* an oxidative burst (Rechnitzer and Kharazmi, 1992; Sahney et al., 2001). Additionally, activation and proliferation of T-cells are both inhibited by direct cleavage of the CD4 receptor and interleukin-2 during assays with purified protease (Mintz et al., 1993). Moreover, a ProA-dependent reduction of the B-cell differentiation factor IL-6 was also observed in cell culture infections (McCoy-Simandle et al., 2011). Even though *in vivo* studies on this topic are rare, ProA was found active against multiple important immune regulators.

Considering these effects on humoral and cellular defense mechanisms, the *L. pneumophila* zinc metalloprotease seems to be especially important during the pathogenesis of Legionnaires’ disease and may represent one of the virulence factors that enable the pathogen to infect multicellular organisms. This also holds true for the interaction of *L. pneumophila* with structural components of the tissue at the site of infection, since ProA contributes to bacterial proliferation and dissemination in human lungs as well as formation and progression of pulmonary damage causing the severe pneumonia-related symptoms in patients. Evaluation of ProA effects in HLTEs as well as guinea pig models revealed significant tissue destruction with inflammation and immune cell infiltration comparable to the histopathology of Legionnaires’ disease (Baskerville et al., 1986; Scheithauer et al., 2021). Occurring lesions demonstrated a correlation between the extent of tissue damage and the protease concentration used (Williams et al., 1987). A prominent histomorphological feature was the significant swelling of alveolar septa due to ProA treatment. These observations can be attributed to structural changes and decomposition of connective tissue fibers at the basal lamina resulting in edema formation (Baskerville et al., 1986; Scheithauer et al., 2021). Early histopathological studies of deceased patients already described an edema-related widening of alveolar septa as a characteristic feature associated with Legionnaires’ disease (Hernandez et al., 1980). Thus, quantification of the alveolar septal thickness represents a suitable indicator for inflammatory processes and the extent of tissue damage in the HLTE model system. As a barrier between individual alveoli during infection, destruction of these septa represents a central step in the development of fibrosis and progression of the disease (Ohta et al., 2012). Accordingly, transmigration and growth studies in treated HLTEs showed reduced spread and proliferation of a *proA* deletion mutant with less invasive bacteria in deeper tissue layers compared to the *L. pneumophila* wild type (Scheithauer et al., 2021). These effects are also in line with earlier observations in guinea pig models (Moffat et al., 1994a).

Altered and finally dissolved alveolar septa are predominantly made up of collagen and elastin fibers. In contrast to the M4 homologue pseudolysin, ProA exhibits no elastase activity (Thompson et al., 1981; Conlan et al., 1986). However, early studies indicated possible collagen degradation by the zinc metalloprotease in azocollagen assays and histological examinations of guinea pig lungs following intranasal administration of ProA (Baskerville et al., 1986; Conlan et al., 1986). Recently, direct proteolysis of human collagen IV, the predominant collagen type of interalveolar septa, was verified *in vitro*. In these assays, highly assembled complexes were rapidly degraded. Likewise, collagen IV antibody staining and quantification of the septal width revealed modification and disintegration of the collagen backbone in HLTEs (Scheithauer et al., 2021). Moreover, activity against human vitronectin, which mediates primarily cell adhesion in pulmonary tissue, was also demonstrated *via in vitro* degradation assays (Hayman et al., 1985; Schvartz et al., 1999; Scheithauer, 2022). Thus, ProA promotes dissemination of *L. pneumophila* by cleavage of different structural host targets, especially disrupting the collagen IV-assembled interalveolar barrier in human lungs. The protease is therefore required in later stages of the intracellular life cycle and the infection.

### 4.3 Sequence homology and X-ray structure analysis classify ProA into the family of M4 zinc metalloproteases with broad substrate specificity

**Figure 2:**
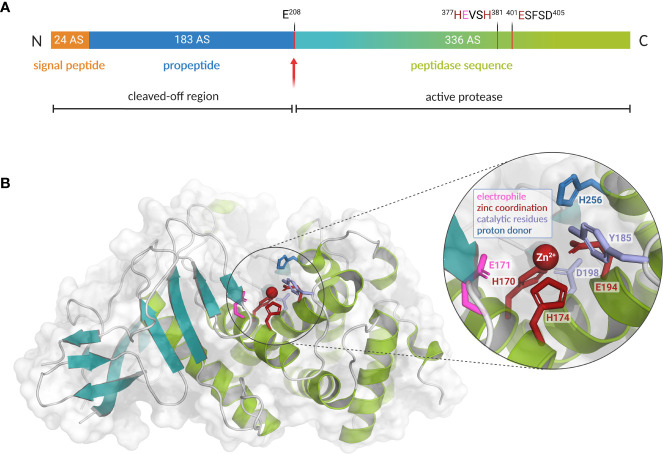
Preproenzyme composition, structure and catalytic center of *L. pneumophila* M4 zinc metalloprotease ProA. **(A)** Schematic overview of the ProA preproenzyme containing a signal- and a propeptide (60 kDa, 543 amino acids). Secretion and autoprocessing at residue Glu 208 results in a 38 kDa mature peptidase unit (teal and green). Important residues for cofactor coordination (red) and proteolytic activity (pink) are highlighted within the zinc-binding motifs. Especially the HExxH motif is highly conserved among the M4 family of TLPs. **(B)** Two domain structure of ProA (PDB code: 6YA1) with N-terminal β-sheets (teal) and C-terminal α-helices (green). A close-up of the active center displays the zinc cofactor as well as amino acid residues for coordination and catalysis (created with PyMOL 2.0 and BioRender.com).

During the stationary phase, expression of ProA is induced *via* the alternative sigma factor RpoS and the response regulator LqsR (Tiaden et al., 2007). The open reading frame (ORF) of ProA comprises a 1629 bp gene sequence, which codes for a translational product with 534 amino acids and 60.8 kDa (Black et al., 1990). The strikingly conserved spatial proximity to the lipA gene of a monoacylglycerol lipase might indicate a common regulation or functional coordination of both gene products (White and Cianciotto, 2019). The inactive ProA precursor is first transported across the inner membrane in a Sec-dependent manner and then further secreted by the T2SS (Hales and Shuman, 1999). In addition to the peptidase unit, the preproenzyme is composed of a 24 amino acids long N-terminal signal peptide and the propeptide with 183 amino acids, making up about a third of the total length (**Figure 2A**). After export, both are cleaved off autocatalytically. The resulting 37.8 kDa mature protease encompasses the C-terminal region of the translational product beginning with a glutamate at position 208 (Moffat et al., 1994b). Its isoelectric point (pI) ranges between 4.20 and 4.42 and the optimum of enzyme activity is dependent on neutral pH values between 5.5 and 7.5. At temperatures of at least 40°C a significant loss of activity occurs, whereas 60°C or more lead to a complete denaturation of the enzyme. Since ProA possesses a central catalytic zinc cofactor, the proteolytic activity is effectively reduced by complexing agents such as EDTA (ethylenediaminetetraacetic acid) or EGTA (ethylene glycol-bis(β-aminoethyl ether)-*N*,*N*,*N*′,*N*′-tetraacetic acid). However, this effect can be reconstituted by the addition of free zinc ions. Interestingly, enzyme activity can also be partly restored using other divalent cations such as Fe^2+^, Cu^2+^ or Mn^2+^ (Thompson et al., 1981; Dreyfus and Iglewski, 1986). In this context, it is noticeable that ProA is expressed less when cultivated in chemically defined minimal medium with limited zinc source compared to complex media (Szeto and Shuman, 1990).

In many infectious bacteria, M4 proteases such as ProA are of particular relevance during pathogenesis. The best-known representative of this enzyme family is thermolysin from *Bacillus thermoproteolyticus*, which is the reason why these endopeptidases are also known as thermolysin-like proteases (TLPs). Characteristically, translation of TLPs results in much longer preproenzymes, which also comprise a signal and a propeptide in addition to the actual peptidase unit. As a chaperone, the latter supports correct folding and, due to its inhibitory function, prevents destructive activity in the bacterial cytoplasm. Autocatalytic processing to the active form only occurs after export to the periplasm. However, the cleaved fragment remains associated with the mature protease until secretion is completed (Yeats et al., 2004; Gao et al., 2010). Due to their extracellular site of action, TLPs are usually involved in the breakdown of foreign proteins and thus contribute to the generation of important nutrients, the bacterial spread in multicellular organisms and the defense against host immune factors (Miyoshi and Shinoda, 2000). All M4 proteases exhibit a rather broad range of substrates and hydrolyze peptide bonds zinc-dependently. Even though sequence identity varies significantly between different members, there is a high level of structural identity, particularly regarding the conserved amino acid residues for hydrolysis and zinc coordination. As part of the active site, the cofactor is located in the interdomain region between N-terminal β-sheets and C-terminal α-helices. It is coordinated by two histidines of the HExxH motif, a glutamate residue of a second zinc-binding motif, and a water molecule (Jongeneel et al., 1989; Jiang and Bond, 1992; Hooper, 1994; Adekoya and Sylte, 2009). These considerable structural homologies imply functional comparability of ProA and other important virulence factors of the TLPs, which also occupy key positions in pathogenesis. Within the M4 enzyme family, ProA shares highest sequence homology of 62.9% with the lasB-encoded elastase pseudolysin from *P. aeruginosa*. Both enzymes show distinct similarities on structural as well as functional level (McIntyre et al., 1991; Poras et al., 2012; Scheithauer et al., 2021). Moreover, efficacy of several competitive inhibitors, such as phosphoramidon, suggests strong conservation regarding substrate binding and conversion (Black et al., 1990). Based on a co-crystallization of pseudolysin and the inhibitor HPI (N-(1-carboxy-3-phenylpropyl)-phenylalanyl-α-asparagine), the structure of ProA was modeled for the first time in 2012. In this context, a highly specific fluorescent substrate was generated for ProA to facilitate the detection of *Legionella* (Poras et al., 2012). Its genomic distribution and immunogenic potential makes the extracellular protease constantly interesting for detection and quantification of the pathogen, and the development of vaccines (Blander and Horwitz, 1989; Poras et al., 2012).

To overcome deficiencies of the elastase-based model, the ProA crystal structure was finally resolved at 1.48 Å by X-ray crystallography in 2020 (**Figure 2B**). Proteolytic and cytotoxic activity was found to be mediated by the glutamate residue Glu 171 of the mature enzyme; experimental studies have already shown that it is essential for substrate conversion and autoprocessing of ProA (Moffat et al., 1994b; Scheithauer et al., 2021). Due to a highly conserved active centre among M4 proteases, with superimposable amino acid residues for zinc binding and catalysis, similar targets are plausible. Nevertheless, significant discrepancies, such as lacking elastase activity of ProA in contrast to pseudolysin, must arise from important differences in peripheral structural elements (Thompson et al., 1981). These variable regions might be involved in substrate recognition or binding. In this context, three flexible, unique loops were identified by comparing ProA to other TLPs. Furthermore, other representatives often possess different numbers or types of cofactors in addition to the common central zinc ion. Calcium is widely distributed in various TLPs and occurs, for example, in pseudolysin and vibriolysin. In these enzymes, it is important for production and processing as well as thermostability (Olson and Ohman, 1992; Eijsink et al., 2011). However, in terms of ProA no additional cofactor could be identified in the native crystal structure apart from the catalytic zinc. According to this, only one zinc ion per protease molecule was detected in atomic absorption spectroscopy (AAS) by Dreyfus and Iglewski in 1986 (Dreyfus and Iglewski, 1986; Scheithauer et al., 2021). Interestingly, de Kreij et al. assumed that differences in the substrate spectrum of M4 proteases are not determined by general sequence or structural differences but are due to a small number of specific amino acids in substrate-binding motifs (de Kreij et al., 2000). The hydrophobic substrate binding pocket S_1_’ is the most important recognition site concerning substrate determination. Site-directed mutagenesis in this region can therefore lead to fundamental changes in the catalytic properties of the proteases. Characteristics of the TLP from *Bacillus stearothermophilus* were already demonstrated to be successfully converted into those of thermolysin by exchanging a single amino acid residue (de Kreij et al., 2000; de Kreij et al., 2001). In pseudolysin, five amino acids from this substrate binding site S_1_’ are also postulated to determine the recognition of the structural human protein elastin (Yang et al., 2015). Four of them can be found at equivalent positions in ProA. Only Phe 129 from pseudolysin is replaced by Met 159 in ProA. If this specific sequence difference is a crucial reason for the lacking elastase activity of ProA or if other factors appear to be more importantly involved in the binding and proteolysis of elastin, remains to be investigated. Nevertheless, structural analyzes showed that Met 159, together with three additional amino acid residues, also narrows the substrate binding pocket of ProA characteristically. Presumably, the zinc metalloprotease is therefore able to expand its binding pocket in the flexible interdomain region through a conformational change upon substrate recognition (Scheithauer et al., 2021). The spatial restriction, however, might hinder binding of stiff and bulky substrates like connective fibers. In contrast to the solid triple helices known from most collagen types, collagen IV forms highly flexible, thin and sheet-like structures at the basal lamina of interalveolar septa. Interestingly, its subunit furthermore exhibits a characteristic curvature 30 nm from the N-terminus, which corresponds perfectly to the obstructive narrowing of ProA’s substrate binding pocket. While the catalytic centre of the protease is less accessible for straight and rigid proteins, this might reflect an adaptation to collagen type IV as a physiologically occurring target (Hofmann et al., 1984; Martin et al., 1985; Ricard-Blum, 2011; Scheithauer et al., 2021).

### 4.4 ProA cleaves and activates bacterial substrates and camouflages *L. pneumophila* by degradation of monomeric flagellin which can be modeled by computational methods

Besides a diverse range of host substrates, ProA shows activity against several *Legionella*-own proteins and thereby influences the intracellular life cycle indirectly (**Figure 1**). The zinc metalloprotease not only regulates these factors by degradation but some T2SS effectors also by specific post-translational activation. The two phospholipases A (PLA) PlaA and PlaC represent virulence-associated factors in the extracellular space, which are activated by ProA *via* processing of a sterically inhibiting disulfide loop (Banerji et al., 2006; Lang et al., 2012; Lang et al., 2017). In this way, *L. pneumophila* most likely prevents itself from enzymatic damage within the cytoplasm. PlaC in particular, but also PlaA, acts as a glycerophospholipid:cholesterol acyltransferase (GCAT), which is expected to hydrolyze various host cell lipids. As a lysophospholipase A (LPLA) and antagonist of the T4SS effector SdhA, which promotes phagosome integrity, PlaA destabilizes the LCV membrane to facilitate the bacterial exit after completed replication (Hiller et al., 2018). Interestingly, ProA expression increases this LPLA activity from PlaA, but abolishes GCAT activity at the same time. In PlaC, however, ProA cleavage induces both PLA and GCAT activity, while the LPLA function appears to be generally independent of the protease (Banerji et al., 2005; Lang et al., 2017). ProA, moreover, processes the bacterial aminopeptidases LapA and LapB, which contribute to the generation of amino acids. LapA exhibits activity against aminopeptides containing leucine, isoleucine, methionine, phenylalanine, valine, aspartate and tyrosine, and is the first representative of secreted aminopeptidases promoting intracellular infection (White et al., 2018). Thus, ProA at least indirectly affects amino acid acquisition and LCV biogenesis *via* its activated bacterial substrates. Nevertheless, ProA can also regulate excess of certain proteins by simple degradation. For example, it reduces free immunogenic flagellin monomers in the extracellular space, which are otherwise recognized by the host immune system (Mascarenhas and Zamboni, 2017; Scheithauer et al., 2022).

**Figure 3:**
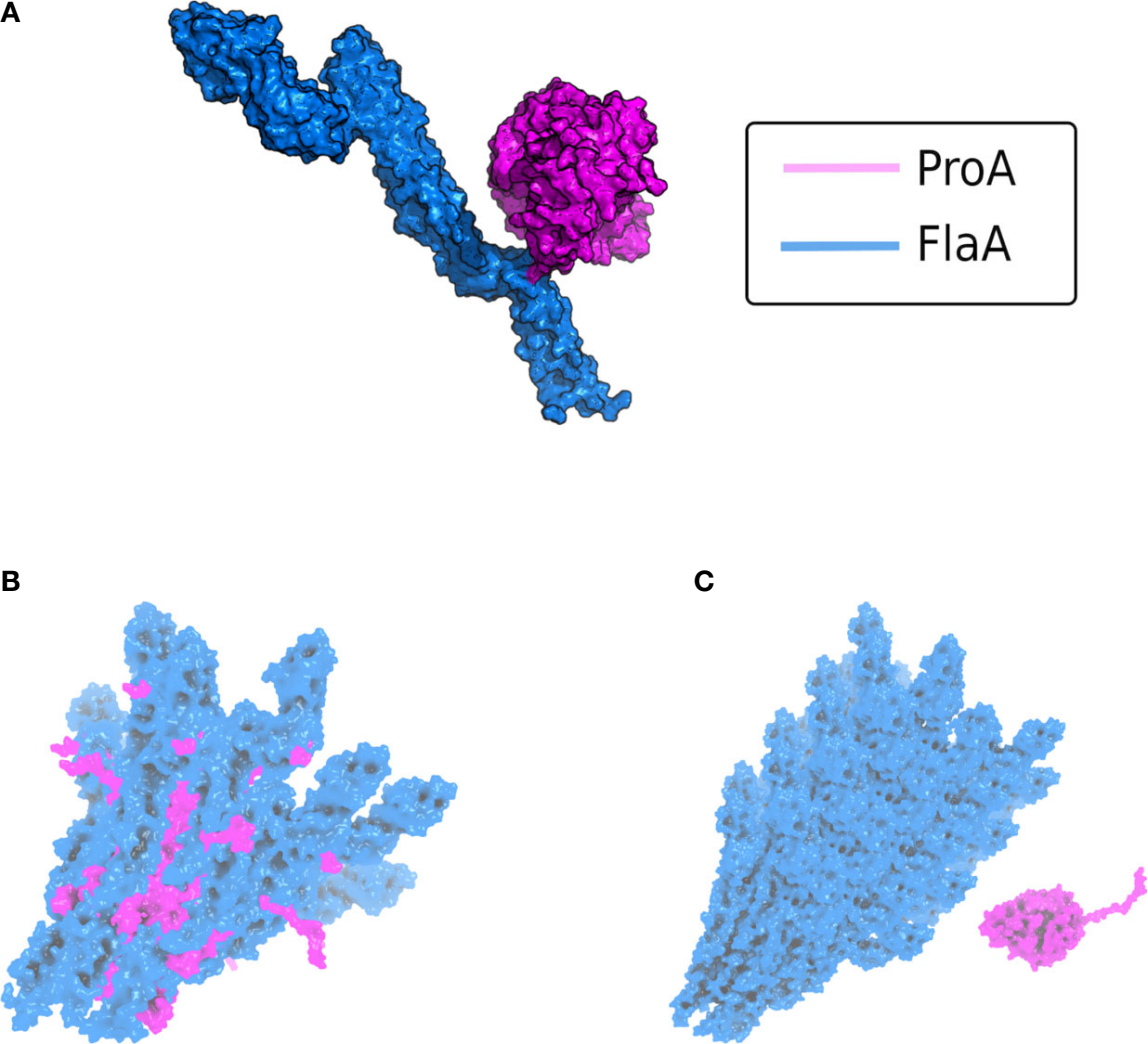
AlphaFold v2.2 multimer modeling of the interaction between *L. pneumophila* ProA and FlaA. **(A)** Complex of the protease ProA in magenta and monomeric FlaA in marine blue. The binding interface with the most contacts is localized within the D_0_ polymerization domain of flagellin. **(B)** Modeling of ProA (magenta) and generic polymerized FlaA (marine blue). The flagellar filament was generated by aligning the predicted *L. pneumophila* FlaA structure on the known Cryo-EM structure of the *P. aeruginosa* filament (PDB ID: 5WK6) (Karagöz et al., 2022). ProA was directly integrated into a deranged flagellar structure, due to steric clashes in the FlaA polymerization region. These clashes were quantified using the repulsive Lennard-Jones (LJ) Energy score from the Rosetta scoring modeling suite according to Alford et al., 2017 and corroborated that this is not a possible protein-protein interaction (Alford et al., 2017). **(C)** Typically assembled filament without access for ProA (ProA placed randomly in vicinity). The conserved cleavage sites of FlaA are hidden within the polymerized flagellar filament.

The finding that the flagella subunit FlaA from *L. pneumophila* is a physiological substrate of the zinc metalloprotease ProA might be of high relevance during the infection of human lung tissue. Interestingly, only monomeric flagellin was shown to be digested, while polymerized FlaA assembling the filament remains stable (Scheithauer et al., 2022). This is important to preserve its crucial functions for the intracellular life cycle. Originally, flagellar expression was associated with the general ability of *L. pneumophila* to initiate infections. In this context, co-regulation with other essential virulence factors represents a central aspect (Pruckler et al., 1995; Bosshardt et al., 1997). FlaA is not expressed while bacterial replication but during the stationary phase to gain access to new host cells (Byrne and Swanson, 1998). Nevertheless, infection of human A549 lung epithelial cells as well as THP-1 macrophages demonstrated significant attenuation of a *flaA* deletion mutant after 24 h and 48 h compared to the *L. pneumophila* wild type (Teruya et al., 2007; Scheithauer, 2022). Correspondingly, a reduced invasion rate was reported, particularly in HL-60 cells (Dietrich et al., 2001; Pereira et al., 2011). Studies of the lung pathogen *P. aeruginosa* revealed that the flagellum stimulates bacterial internalization into the host cell *via* opsonin-independent phagocytosis (Mahenthiralingam and Speert, 1995). Nevertheless, extracellular and unassembled FlaA triggers the immune response upon receptor recognition by the host. Interestingly, both TLR5 stimulation and proteolytic degradation exclusively occur with monomeric flagellin. Thus, recognition by receptor and protease is assumed to be mediated *via* a non-exposed part of FlaA located within the polymerized flagellum. It was already published that TLR5 binding especially involves specific amino acid residues of the highly conserved D_1_ domain of flagellin but also of the polymerization domain D_0_ (Forstnerič et al., 2017; Song et al., 2017). The *L. pneumophila* zinc metalloprotease most likely exhibits a similarly located recognition site. By using AlphaFold v2.2 multimer modeling, we were able to generate a structure of the ProA-FlaA complex with a high score of prediction quality referring to the interface between both proteins (**Figure 3A**). Indeed, this binding interface is localized within the D_0_ polymerization domain of FlaA strengthening our assumption that ProA is only able to degrade flagellin monomers since an assembly of the subunits will cover the protease cleavage sites. By aligning the *L. pneumophila* FlaA structure with the known filament Cryo-EM structure of *P. aeruginosa*, we also modeled polymeric flagellin (Wang et al., 2017; Karagöz et al., 2022). The integration of ProA (magenta) into the multimer prediction resulted in a deranged flagellar structure (marine blue) with steric clashes indicating an impossible protein-protein interaction (**Figure 3B**). This becomes particularly clear when compared with the typically assembled filament, where ProA has no access to the flagellin cleavage site (**Figure 3C**). This lead to the conclusion that ProA is not able to attack the intact surface of a polymerized flagellum but has immunomodulatory potential by degradation of free FlaA.

Immune evasion by the protease was already elucidated *via* a reporter-based TLR5 stimulation assay. Purified and active ProA demonstrably abolished the receptor activation caused by monomeric flagellin. Moreover, continuative experiments displayed diminished recognition of a *flaA* deletion mutant but increased values with a *proA* deletion strain compared to the *L. pneumophila* wild type using bacterial suspensions as well as HLTE infection supernatants (Scheithauer et al., 2022). Conclusively, ProA leads to a significant reduction of flagellin-dependent TLR5 stimulation in a physiological human background. Similar activity and effects were described in detail regarding the homologous pseudolysin and the alkaline protease ArpA, both from *P. aeruginosa*. In an *arpA*-deficient mutant, TLR5 activation is increased 100-fold compared to the wild type, indicating responsibility for a complete degradation of exogenous flagellin (Bardoel et al., 2011; Casilag et al., 2016). In pulmonary tissue, TLR5 is expressed particularly by alveolar macrophages, but also by respiratory epithelial cells, type II pneumocytes, neutrophils, plasma and dendritic cells (Honko and Mizel, 2005; Shikhagaie et al., 2014). Activation of this Toll-like receptor plays a crucial role in fighting legionellosis, which is why people with a TLR5^392STOP^ polymorphism suffer from a significantly enhanced susceptibility to Legionnaires’ disease (Hawn et al., 2003). In this case, flagellin-mediated signaling is disrupted by an alteration in the ligand-binding domain, resulting in a decreased innate immune response of the lung epithelium. The cleavage of free FlaA by ProA hence may represent a major advantage for immune evasion and thus for pathogen proliferation (**Figure 1**). One of the most important pro-inflammatory factors that are induced in a TLR5-dependent and therefore FlaA-dependent manner is IL-8 (Yu et al., 2003; Schmeck et al., 2007; Im et al., 2009). Studies have already shown that IL-8 is also the most common messenger in the supernatant of *Legionella*-infected macrophages (McCoy-Simandle et al., 2011). The chemokine contributes to the recruitment of neutrophils and other immune cells that are crucial for elimination of the pathogen (Tateda et al., 2001; Hawn et al., 2007; Mascarenhas et al., 2015). Additionally, TLR5-mediated recognition of flagellin *via* NF-κB induces TNF-α production in alveolar macrophages. TNF-α is a multifunctional signaling factor, which provokes apoptosis of infected host cells and thus reduces proliferation of *L. pneumophila* in the lungs (Blanchard et al., 1988; Kawamoto et al., 2017). *Pseudomonas* flagellin also triggers the expression of cytokines like MIP-2, IL-17 and IL-22 and antimicrobial peptides such as β-defensin 2 or CRAMP (cathelin-dependent antimicrobial peptide) in a mouse model (Yu et al., 2010). Eventually, FlaA is not only recognized extracellularly by TLR5 on the host cell surface but also detected in the cytoplasm by the Naip5/Nlrc4 inflammasome. Since ProA is likewise translocated from the LCV, it is conceivable that the protease cleaves cytosolic flagellin in order to reduce the inflammasome stimulation. For future works, alteration of pro-inflammatory mediators in downstream pathways should be examined, since already identified targets of ProA include a wide range of host factors.

## 5 Macrophage infectivity potentiator (Mip)

### 5.1 Peptidyl-prolyl-*cis/trans*-isomerase Mip binds collagen and enables bacterial transmigration across tissue barriers

The *L. pneumophila* membrane-associated surface protein Mip was the first genetically identified virulence factor of *L. pneumophila* (Cianciotto et al., 1989; Engleberg et al., 1989). Deletion of the respective gene results in reduced intracellular replication in human alveolar macrophages and protozoa (Cianciotto et al., 1989; Cianciotto and Fields, 1992; Fischer et al., 1992). Mip (24 kDa) possesses an N-terminal signal sequence which is cleaved off while the protein is transported through the cytoplasmic membrane (Wintermeyer et al., 1995). The basic protein (pI 9.8) forms a stable homodimer, and the 2.4 Å crystal structure revealed that each monomer consists of a N-terminal dimerization module, a long connecting α-helix (α3) and a C-terminal peptidyl-prolyl-*cis/trans*-isomerase (PPIase) domain (Riboldi-Tunnicliffe et al., 2001; Helbig et al., 2001; Köhler et al., 2003; Galka et al., 2008). The fold of the C-terminal domain (residues 100-213) shows high homology to the human FK506-binding protein 12 (FKBP12) (Schmidt et al., 1996; Fischer et al., 1998), and the macrolides FK506 or rapamycin efficiently inhibit the PPIase activity of the respective proteins (Schiene-Fischer et al., 2013). Further Mip targeting inhibitors such as cycloheximide, pipecolic acid, as well as non-immunosuppressive FK506 derivatives independently corroborated the observation of moonlighting activities of Mip in fundamental processes of infection (Scheuplein et al., 2020; Rasch et al., 2015; Rasch et al., 2014; Juli et al., 2011; Pomplun et al., 2018; Ünal and Steinert, 2014).

Nuclear magnetic resonance (NMR) solved the solution structure of free Mip^77-213^ and the Mip^77-213^-rapamycin complex, and comparisons with the structures of free FKBP12 and the FKBP12-rapamycin complex suggested an identical binding mode for both proteins (Ceymann et al., 2008). Molecular dynamics simulations of the Mip dimer yielded two different correlation times for the two domains and thus confirmed the independence of the domain motions. Thus, mediated by a hinge in the long α-helix, both FKBP domains of the dimerized Mip appear highly flexible for cooperative binding of potential target structures (Horstmann et al., 2006).

**Figure 4:**
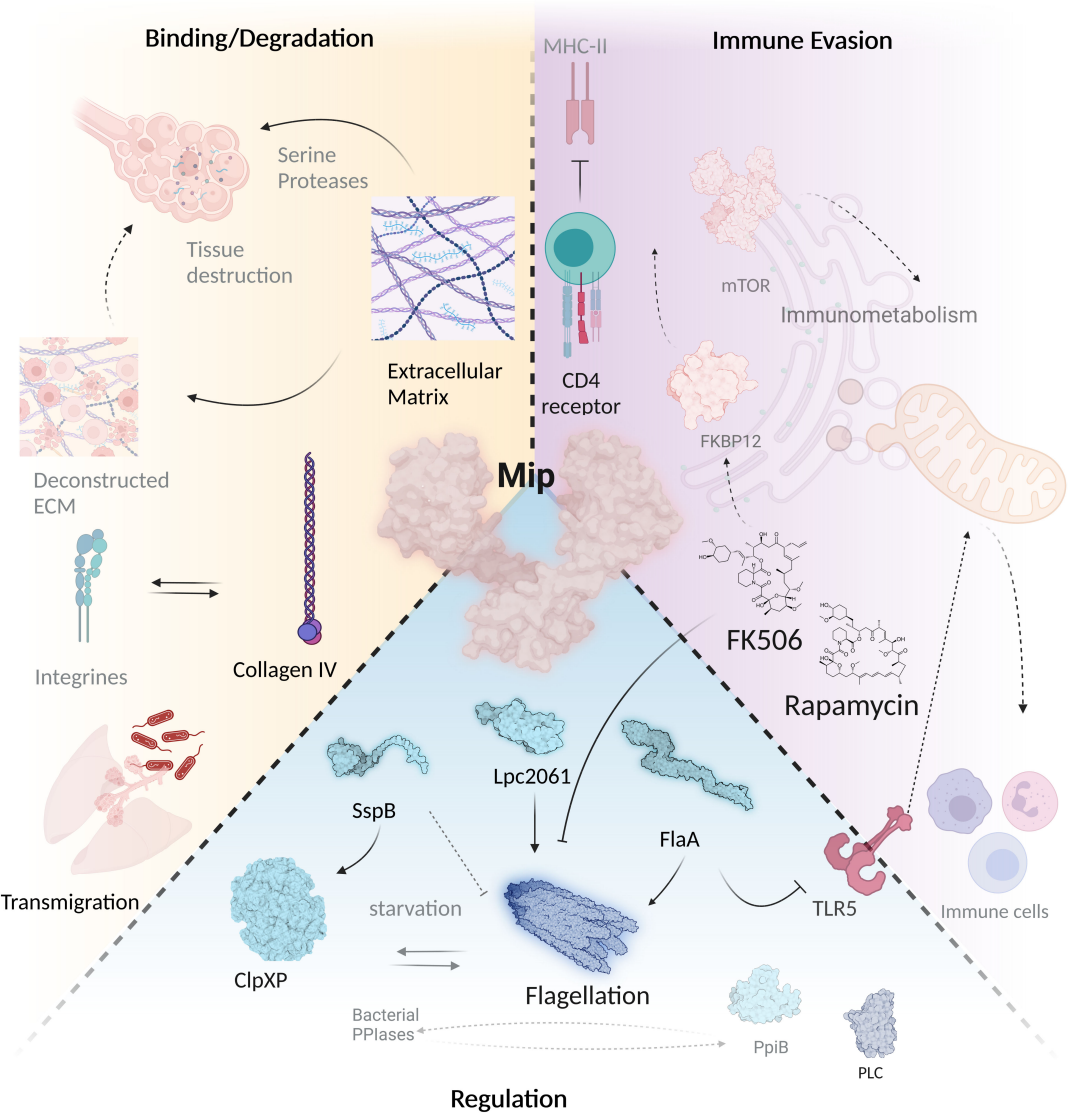
Mip mediates virulence regulation, host tissue degradation and immune evasion. The virulence factor Mip interferes with a wide variety of infection-associated processes *via* different interaction partners. During Legionnaires’ disease, it is important that bacteria migrate through different tissue barriers. This is facilitated by binding of Mip to collagen IV, which is subsequently degraded by a serine protease. Mip binds to SspB, Lpc2061 and FlaA, and promotes flagellation by its PPIase domain that can be inhibited by FK506 and rapamycin. Binding of FK506 and rapamycin to PPIases modulates immunometabolism and interferes with bacterial immune evasion. Mip regulates stress response and infectivity in bacteria together with other PPIases and promotes Phospholipase C (PLC) activity (created with BioRender.com).

PPIases of pathogens such as *Burkholderia*, *Chlamydia*, *Clostridium*, *Neisseria*, *Klebsiella* and others are generally involved in a broad spectrum of phenotypes including virulence, metabolism, and multiple stress responses (Lundemose et al., 1991; Norville et al., 2011; Ünal and Steinert, 2014; Ünal et al., 2018; Ünal et al., 2019; Christodoulides, 2022; Iwasaki et al., 2022). Likewise, the PPIase Mip of *L. pneumophila* contributes to infection, collagen binding, phospholipase C-like activity, transmigration across tissue barriers, nematode colonization, surface translocation, and growth at suboptimal temperature (**Figure 4**) (DebRoy et al., 2006; Ünal et al., 2011; Rasch et al., 2019; Rasch et al., 2016). Although previous studies employing genetic and biochemical methods together with different infection models revealed that Mip impacts the course and outcome of infection on multiple levels (Wintermeyer et al., 1995; Köhler et al., 2003; Wagner et al., 2007; Söderberg and Cianciotto, 2008), our knowledge about binding partners or substrates of Mip remained very limited. In contrast, the structural understanding of Mip improved steadily. Site-specific mutagenesis of highly conserved amino acid residues within the FK506-binding pocket, in which Asp 142 was replaced by leucine and Tyr 185 by alanine, revealed pronounced loss of PPIase activity of the purified recombinant protein *in vitro* (residual activity 6.2% for the D142L mutant and 2.0% for the Y185A mutant). Surprisingly, wild type phenotypes in infection studies with *A. castellanii* or human macrophage-like cell lines were observed, when the same site specifically mutated variants of *mip* were used to complement *L. pneumophila mip*-negative mutants (Wintermeyer et al., 1995). This suggests that either the residual enzymatic activity of the mutated Mip was still sufficient for PPIase-dependent phenotypes, or additional properties other than the PPIase activity are important during intracellular infection. Strikingly, guinea pig infections with *L. pneumophila* strains expressing Mip variants, that were unable to dimerize or had a low PPIase activity, were significantly attenuated (Köhler et al., 2003). This was in good agreement with the observation that Mip-deficient bacteria were found to be attenuated and unable to disseminate systemically in guinea pigs (Wagner et al., 2007).

The different consequences of a reduced PPIase activity in monocellular infection models and guinea pig infections indicated additional activities of Mip in the course of the more complex infection of mammalian tissues. This interpretation was further corroborated when transwell assays with *L. pneumophila* and recombinant *Escherichia coli* HB101 strains revealed that Mip enables these bacteria to transmigrate across a barrier of NCI-H292 lung epithelial cells and extracellular matrix (NCI-H292/ECM barrier). Further evidence for an extracellular function of Mip resulted from the observation that Mip binds to collagen I-VI (**Figure 4**) (Wagner et al., 2007). Especially interesting is the fact that the best binding of Mip was determined for collagen IV, which is known to be the prevalent collagen type in the human lung (Gelse et al., 2003). Mip binds to a surface-exposed sequence in the NC1 domain of the collagen IV α1 chain, and a corresponding collagen IV-derived peptide (IPPCPSGWSSLWI; P290) co-precipitated with Mip and competitively inhibited the Mip-collagen IV binding (Ünal et al., 2011). Moreover, P290 efficiently inhibited transmigration of *L. pneumophila* across a barrier of NCI-H292 lung epithelial cells and extracellular matrix. This significantly reduced transmigration was comparable to the inefficient transmigration of PPIase-negative Mip mutant or rapamycin-treated *L. pneumophila*. Based on NMR data and docking studies, a model for the mode of interaction of P290 and Mip was developed. The amino acid residues of the hydrophobic cavity of Mip, D142 and to a lesser extent Y185, were identified to be part of the interaction surface (Wintermeyer et al., 1995).

### 5.2 Computational methods allow instructive interpretations of Mip interactions with stringent starvation protein SspB, hypothetical protein Lpc2061, and flagellin FlaA

**Figure 5:**
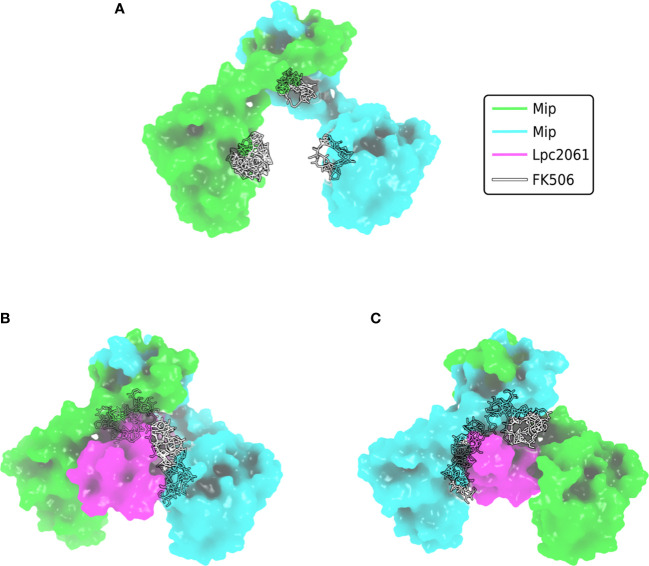
Docking of FK506 to Mip and competitive inhibition of Lpc2061 binding. **(A)** Docking of FK506 (grey) to the Mip homodimer (green and cyan) results in binding to the dimerization and the two PPIase domains. **(B)** Lpc2061 (magenta) binding to Mip is inhibited by FK506, since the inhibitor blocks this triangle. **(C)** Overlapping binding regions of Lpc2061 and FK506 after rotation by 180°. Molecular docking was performed using DiffDock (Corso et al., 2022).

Although being the first identified virulence factor of *L. pneumophila*, it remained largely unknown for a long time, how Mip exerts its diverse functions. A recent interactomic approach, however, paved the way for more straight forward functional studies, since the stringent starvation protein B (SspB, LPC_0434), hypothetical protein Lpc2061 (LPC_2061) and flagellin (FlaA, LPC_0756) were identified as *in vivo* bacterial interaction partners of Mip (**Figure 4**) (Karagöz et al., 2022). Addition of the macrolide FK506 in co-immunoprecipitation assays revealed that only Lpc2061, but not SspB or FlaA, requires the C-terminal PPIase binding pocket of Mip for interaction. Recent machine learning models suggest the docking sites of FK506 to the Mip homodimer and indicate why additional binding of Lpc2061 is inhibited (**Figure 5**) (Corso et al., 2022). The macrolide FK506 occupies the basis of the α-helix at the dimerization region and the cavities of both PPIase domains (**Figure 5A**). Since Lpc2061 requires this triangle for Mip binding, competitive inhibition by FK506 appears to be the most likely mechanism (**Figures 5B, C**).

**Figure 6:**
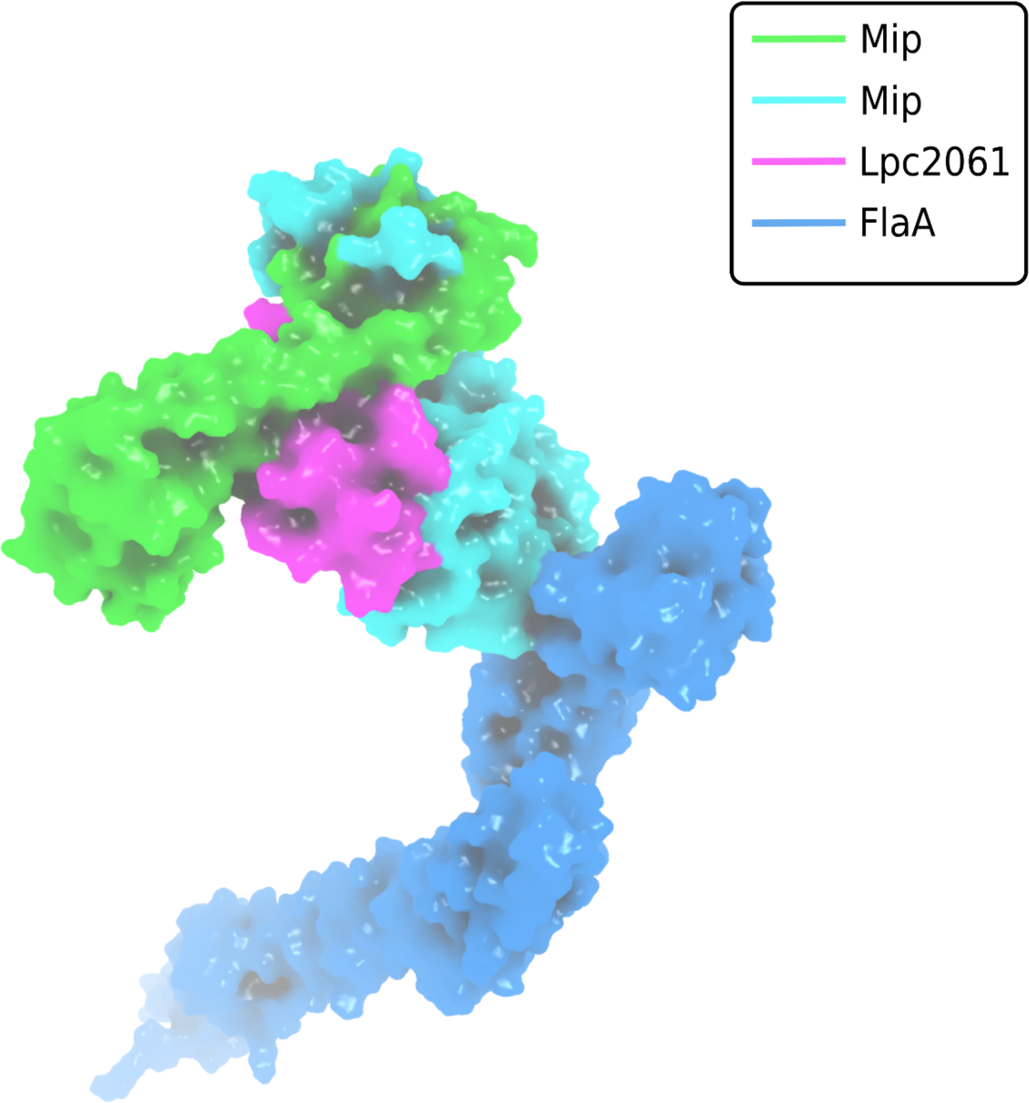
Surface plot visualization of the tripartite complex of Mip, FlaA and Lpc2061. Modeling was perfomed by docking of FlaA (marine blue) to the best pose of Lpc2061 (magenta) and the Mip homodimer (green and cyan). While Lpc2061 binds to the Mip dimerization region, monomeric FlaA interacts with the C-terminus of the PPIase domain. This tripartite complex was found to be more stable than the binary Mip-Lpc2061 interaction (Karagöz et al., 2022).

The finding that not all interactions were negatively influenced by FK506 was not surprising since several of the virulence and fitness functions of Mip are not related to enzymatic catalysis, but rather to moonlighting activities in the host (Rasch et al., 2014; Rasch et al., 2015; Ünal et al., 2011). Evaluation of the biochemical data and computational predictions of the respective interactions with Mip consistently suggested that SspB is the strongest binder, followed by Lpc2061 and FlaA. Interestingly, molecular dynamic simulations predicted an increased stability for the tripartite interaction of Mip, Lpc2061 and FlaA compared to the Mip-Lpc2061 binary interaction (**Figure 6**).

*L. pneumophila* strains expressing Mip variants with single amino acid substitutions or N-terminally truncated monomers revealed that the dimerization region and the amino acid residue Y185 of Mip are required for the binding of Lpc2061, which is strengthened by FlaA. The binding of SspB occurs independently of the tested Mip variants and is not influenced by the presence of FlaA. Modeling of the interaction partners and global docking with Mip suggested non-overlapping binding interfaces, and molecular dynamic simulation predicted, consistent with the biochemical data, an increased stability for the tripartite interaction of Lpc2061, Mip, and FlaA (Karagöz et al., 2022).

The observed interactions of Mip with the respective bacterial binding partners raise questions regarding their functional implications. SspB homologues of *E. coli* (52% identity) and *Pseudoalteromonas* spp. (62% identity) are well known dimeric adaptor proteins, which increase the rate at which ssrA-tagged substrates are degraded by tethering them to the ClpXP protease (Levchenko et al., 2000; Yin et al., 2021). Respective ClpXP deletion mutants of *Salmonella enterica* Serovar Typhimurium exhibit overproduction of the flagellar protein, a fourfold increase in the rate of transcription of *fliC*, and a hyperflagellated phenotype (Tomoyasu et al., 2002). Whether Mip intercepts SspB and by this means downregulates the ClpXP-dependent repression of the flagellar regulon in *L. pneumophila* remains to be elucidated. The function of the hypothetical protein Lpc2061 with structural homology to glycoside hydrolases also remains to be investigated. The interaction of Mip and FlaA is especially interesting, since the bacterial PPIase was demonstrated to influence flagellation and motility of *L. pneumophila* (Karagöz et al., 2022).

### 5.3 Mip interactions promote flagellation and bacterial motility which is inhibited by FK506

Recent results demonstrated Mip as a binding partner of FlaA and amplifier of *L. pneumophila* flagellation and motility (Karagöz et al., 2022). Moreover, this phenotype was positively modulated by the Mip interaction partner Lpc2061 (**Figure 4**). Since FlaA and SspB are both expressed during starvation periods in the post-exponential phase of *L. pneumophila*, it is of future interest to analyze if the Mip-SspB binding also influences this interaction (Appelt and Heuner, 2017).

Biochemical quantifications of FlaA preparations of *L. pneumophila* wild type strains and mutants demonstrated that wild type Mip promotes flagellation of *L. pneumophila* and the yield of FlaA. *L. pneumophila* strains expressing the Mip^(Y185A)^ or the monomeric Mip^(77-213)^ variant, which bind less Lpc2061, were less flagellated and yielded less FlaA. Also, FK506 treatment resulted in a lower FlaA yield and reduced motility. In accordance with biochemical results showing that FlaA and Lpc2061 mutually reinforce their binding to Mip, which was also suggested by molecular dynamic simulations, it was described that the binding regions of Mip in Mip-Lpc2061 interaction positively influence flagellation (Karagöz et al., 2022).

For a long time it is known that Mip and the major subunit FlaA both positively affect the early phase of infection of eukaryotic host cells such as amoebae and macrophages (Dietrich et al., 2001; Hammer et al., 2002). More recent work suggests that certain effects of Mip or FlaA on *L. pneumophila* pathogenicity are mediated or regulated by their interaction (Karagöz et al., 2022). However, whether or how Mip assists in the flagellar assembly or regulation remains to be elucidated.

## 6 Conclusion, open questions and future perspectives in drug research

Infection and disease progression largely depend on the outcome of protein-protein interactions between pathogen and host. Since *L. pneumophila* evolved in the aquatic environment and not in humans, ProA and Mip should also have ecological implications. Both virulence factors are important for the replication in certain protist species. Nevertheless, the question remains open, how ProA and Mip became able to recognize target structures like e. g. collagen, which are characteristic for higher eukaryotes. One hypothesis is that an intimate coexistence with native metazoan species as hosts, vectors, or reservoirs lead to the acquisition of eukaryotic protein domains, the development of collagen-binding motifs, and immune evasion strategies (Best and Abu Kwaik, 2018). However, it is also conceivable that most of the human targets represent accidental ProA substrates due to a broad specificity of the protease. Thus, studies clarifying the role of ProA and Mip in multicellular hosts of natural aquatic habitats such as nematodes will be of particular interest. In this context, it appears also promising to apply interactomic approaches to identify further host targets of Mip.

The virulence factors ProA and Mip both target *L. pneumophila* FlaA and the host ECM protein collagen by degradation or binding, respectively (Scheithauer et al., 2021; Scheithauer et al., 2022; Karagöz et al., 2022; Köhler et al., 2003; Wagner et al., 2007). On the phenotypic level, both virulence factors (i) promote bacterial transmigration in tissue, (ii) regulate virulence factors including flagellation, and (iii) influence immune evasion including immunometabolism. This raises the question to what extent the activities of Mip and ProA are cooperative and coordinated in a functional virulence network. Previous work with metabolically labeled ECM revealed that Mip-positive bacteria degrade ECM proteins, whereas Mip-negative bacteria or pure recombinant Mip protein do not cause degradation (Wagner et al., 2007). Moreover, the degradation of ECM could be inhibited by the serine protease inhibitors Pefabloc SC and PMSF, although the Mip PPIase activity was not affected by this treatment (Köhler et al., 2003; Wagner et al., 2007). These observations demonstrated that Mip does not degrade the ECM through an own proteolytic activity and suggested that an additional serine protease activity is required. More recent data revealed that ProA mediates tissue damage in HLTEs by degradation of collagen IV in the basal lamina and the cell adhesion protein vitronectin (Ünal et al., 2011; Scheithauer, 2022). If the effects of Mip and ProA are directly linked, and how a yet not identified serine protease of *L. pneumophila* or the host cells operates in a concerted way, remains to be elucidated. Nevertheless, a pharmacological approach using FK506 or rapamycin in the ECM degradation assay suggested that the PPIase activity is required for the observed proteolysis (Wagner et al., 2007).

Further functional questions arise from the identified tripartite interaction of FlaA, Lpc2061 and Mip and from the degradation of monomeric FlaA by ProA, which reduces the TLR5-mediated immune response. To avoid bacterial clearance by the immune system *L. pneumophila* seems to follow the strategy to either polymerize FlaA into flagella, or to minimize the amount of exogenous monomeric FlaA (Forstnerič et al., 2017; Song et al., 2017). Whether, and if so how, Mip assists FlaA polymerization or ProA degradation is not known. But since Mip-negative *L. pneumophila* strains are less flagellated, yield less FlaA and are non-motile, we hypothesize a coordinated contribution of Mip and ProA to avoid free FlaA monomers.

Since computational approaches have already been successfully applied for the characterization of ProA, Mip and their respective binding partners, it seems possible to also model protein-drug interactions. The fast developing machine learning approaches together with molecular dynamics simulations, docking and X-ray diffraction crystallography or Cryo-EM have the potential to create novel scoring functions to anticipate ligand-binding affinity with high predictive power. By using regression methods and deep learning models trained for ensemble prediction, it is e.g. possible to scan the scoring function space and to gain insight into protein-ligand energetics. Since we currently witness rapid progress due to the application of deep learning methods to predict the 3D structures of proteins, we may soon be able to generate refined protein sociological studies which include options for interference by drugs.

## Author contributions

All authors listed have made a substantial, direct, and intellectual contribution to the work and approved it for publication.
